# Relationship between metastasis and second primary cancers in women with breast cancer

**DOI:** 10.3389/fonc.2022.942320

**Published:** 2022-09-29

**Authors:** Chaofan Li, Mengjie Liu, Jia Li, Xixi Zhao, Yusheng Wang, Xi Chen, Weiwei Wang, Shiyu Sun, Cong Feng, Yifan Cai, Fei Wu, Chong Du, Yinbin Zhang, Shuqun Zhang, Jingkun Qu

**Affiliations:** ^1^ Department of Oncology, the Second Affiliated Hospital of Xi’an Jiaotong University, Xi’an, China; ^2^ Department of Radiation Oncology, the Second Affiliated Hospital of Xi’an Jiaotong University, Xi’an, China; ^3^ Department of Otolaryngology, the Second Affiliated Hospital of Xi’an Jiaotong University, Xi’an, China

**Keywords:** breast cancer, metastasis, second primary cancers, XGBoost algorithm, standardized incidence ratio, standardized mortality ratio

## Abstract

**Background:**

Breast cancer (BC) survivors have an increased risk of developing second primary cancers (SPCs); however, it is still unclear if metastasis is a risk factor for developing SPCs. Usually, long-term cancer survivors face an increased risk of developing SPCs; however, less attention has been paid to SPCs in patients with metastatic cancer as the survival outcomes of the patients are greatly reduced.

**Methods:**

A total of 17,077 American women diagnosed with breast cancer between 2010 and 2018 were identified from Surveillance, Epidemiology, and End Results (SEER) database and were included in the study. The clinical characteristics, standardized incidence ratio (SIR), standardized mortality ratio (SMR), and patterns of SPCs in BC patients with no metastasis, regional lymph node metastasis, and distant metastasis were investigated. Kaplan-Meier method was used to compare the prognosis of BC patients after developing SPCs with different metastatic status. XGBoost, a high-precision machine learning algorithm, was used to create a prediction model to estimate the prognosis of metastatic breast cancer (MBC) patients with SPCs.

**Results:**

The results reveal that the SIR (1.01; 95% CI, 0.99–1.03, p>0.05) of SPCs in non-metastasis breast cancer (NMBC) patients was similar to the general population. Further, patients with regional lymph node metastasis showed an 8% increased risk of SPCs (SIR=1.08, 95%CI, 1.05–1.11, p<0.05), and patients with distant metastasis had a 26% increased risk of SPCs (SIR=1.26, 95%CI, 1.16–1.37, p<0.05). The SIR of SPCs in all patients below the age of 40 was the highest, which decreased with age. Patients with poorly differentiated cancers, large tumor size, and late N stage had an increased risk of SPCs. However, an increase in SIR of SPCs was observed in distant MBC patients, even at the early T1 (SIR=1.60, 95% CI, 1.22–1.98, p<0.05) and N1 (SIR=1.27, 95% CI, 1.10–1.44, p<0.05) stage. An increase in the SIR of SPCs was observed in patients with triple-negative BC, and the SIR of SPC increased with metastasis development in BC patients with luminal A subtype. The peak of SPCs risk occurrence was earlier in MBC patients (4-6 months and 10 months) compared to NMBC patients (12 months). The effect of metastasis on the prognosis of SPCs patients was dependent on the type of SPCs. Meanwhile, the XGBoost model was created to predict the 3-year (AUC=0.873) and 5-year survival (AUC=0.918) of SPCs in MBC patients.

**Conclusions:**

Our study provides novel insight into the impact of metastasis on SPCs in BC patients. Metastasis could promote the second primary tumorigenesis which further increased cancer-related deaths. Therefore, more attention should be paid to the occurrence of SPCs in MBC patients.

## Introduction

Globally, breast cancer (BC) has surpassed lung cancer as the most common cancer (1). Due to the advancement in BC treatment, the survival of BC patients has significantly improved. However, BC survivors have a higher risk of developing second primary cancers (SPCs) at different sites (2). The increased risk of developing SPCs could be due to shared etiology, genetic susceptibility, environmental and lifestyle factors, and long-term side effects of BC treatment (3). However, limited information is available if metastasis increases the risk of SPCs development in BC patients.

Metastasis is the main cause of death in BC patients (4). The 5-year overall survival rate of non-metastasis breast cancer (NMBC) patients is over 95%; however, the survival rate decreases to 85% for patients with regional axillary lymph node metastasis (5), which further reduces to only 25% in the case of distant metastasis (6). The most commonly affected distant organs are bone, lung, liver, and brain (4). Approximately 75% of these patients develop bone metastasis (7), which usually is the first sign of cancer recurrence, and the 5-year overall survival rate is 22.8% (8). The lung is the second most frequent site of BC metastasis, with a 5-year overall survival rate of 16.8% (9). The occurrence of liver metastasis is slightly lower than lung metastasis, and the 5-year overall survival rate is poor (not over 9%) (10). About 15–30% of women with metastatic BC may develop brain metastases, and the prognosis is extremely poor, with survival ranging from 2–23 months post diagnosis (11). Further, the long-term survivors face an increased risk of SPCs (12). Despite the advancement in medical technology, the involvement of metastasis in the risk of developing SPCs has received less attention. Hence, it is important to understand the role of metastasis in the development of SPCs.

Generally, survival time is the most important concern for patients with BC, specifically post the diagnosis of metastasis and SPCs. Further, there is a lack of an accurate prediction model for metastatic breast cancer (MBC) patients with SPCs. Nomogram is the most used model to predict the survival rate of patients; however, its accuracy rate is only about 70% (13, 14). Hence, a more accurate and robust predictive model is needed. Recently, machine learning methods have been used to create an artificial intelligence (AI) model to predict the survival of cancer patients, which significantly increases the accuracy of prediction (15). However, traditional machine learning has some recognized flaws. For example, Support Vector Machines (SVMs) fail to handle large volumes of samples and variables, k-Nearest Neighbors (KNNs) are difficult to interpret, and the decision trees are easy to train quickly but are not complex enough (16). Extreme Gradient Boosting (XGBoost) algorithm uses a technique called “feature subsampling,” used in random forests to prevent overfitting and was created iteratively to minimize loss of function. This approach allows the algorithm to excel in many areas but has rarely been used to predict the prognosis of cancer patients. We used the inter-model comparison, and the results revealed that XGBoost demonstrated excellent performance in resolving the problems associated with prognosis.

In this study, we examined the association between metastasis and SPCs in BC patients using data retrieved from Surveillance Epidemiology and End Results (SEER) database. The incidence, mortality, survival, and patterns of SPCs in different metastasis status were investigated. Further, a high-precision AI model was created to predict the survival of MBC patients after SPCs diagnosis. These results provide insights into the SPCs of BC patients, which aids in improving the long-term follow-up for SPCs diagnosis, especially in distant MBC patients.

## Materials and methods

### Data source and study design

**Figure 1:**
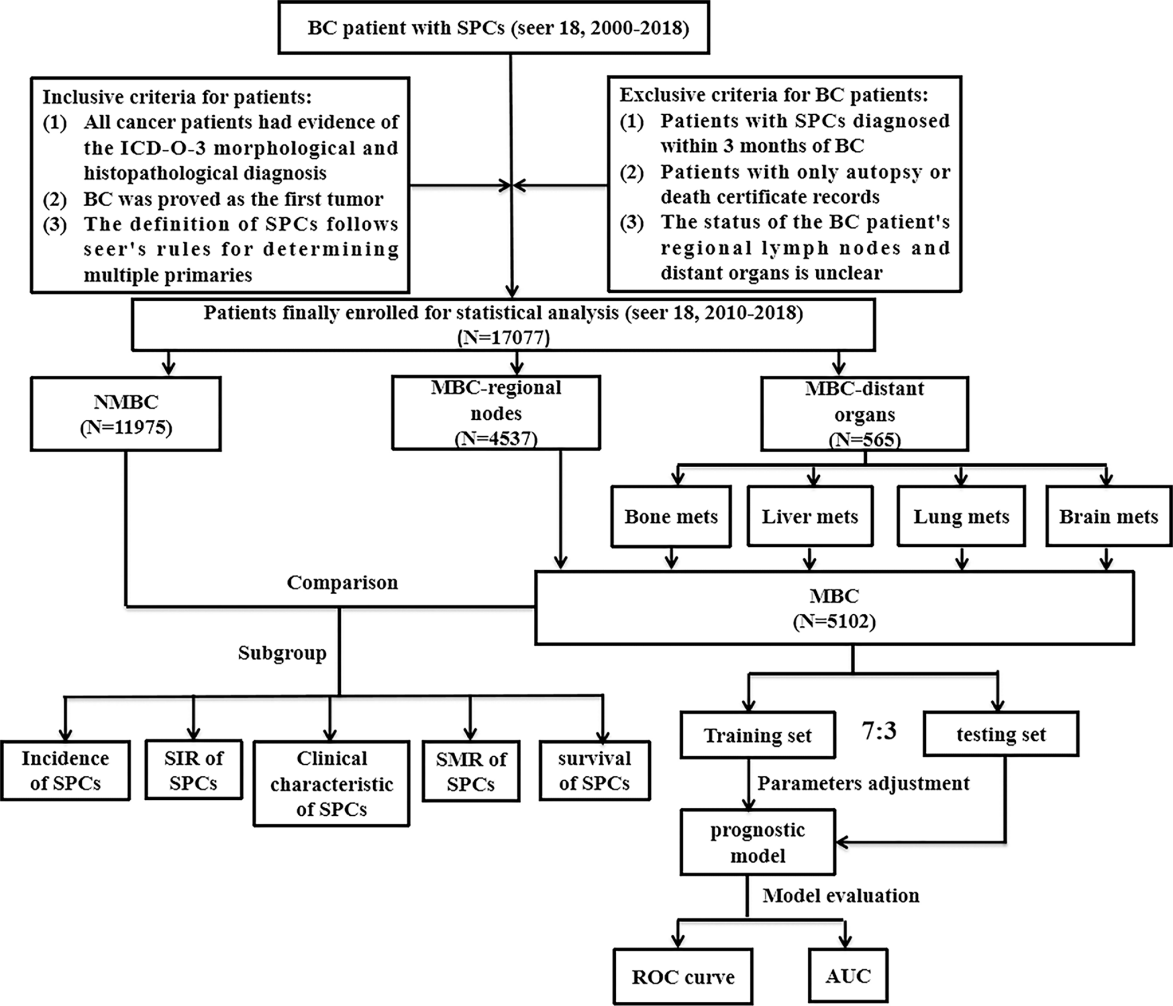
The flowchart shows the process of conducting the study and statistical analysis.

The workflow of our study design and analysis is shown in **Figure 1**. SEER database collects information on cancer patients representing 34.6% of the U.S. population. The information on distant metastasis has been available since 2010; hence the data used for the analysis in this study were retrieved from the SEER database [SEER 18 Regs Study Data, (2000-2018 changes); version 8.3.9]. The study was approved by the Institutional Review Committee (IRC) of the Second Affiliated Hospital of Xi’an Jiaotong University. The IRC granted a waiver for informed consent since the data was obtained from publicly available databases and did not reveal any identifiable information. From this database, data on women with BC and any SPCs were collected. BC has been proved to be the first primary tumor. All cancer patients included in the study had evidence of the International Classification of Cancer Diseases Edition III (ICD-O-3) morphological and histopathological diagnosis. SPCs were identified using the following SEER rules: 1) if the histological type of the new lesion is same as the original lesion and have occurred at the same time within two months, the lesion would identified as a single lesion and not as a primary new lesion; 2) if the histological type of the new lesion is different from that of the original lesion, and lesion occurred at the same site and the same time (within 2 months), this lesion would identified as the new primary tumor; 3) the presence of an achromatic lesion at the same site (2 months or more after the initial diagnosis) would identified as a new primary tumor regardless of histological type, unless the diagnosis reveals a metastatic lesion; 4) new lesions at different sites with the same or different histological types should always be considered as new primary tumors, unless they are clearly metastatic; 5) for paired organs, only one histological type of bilateral synchronous tumor would be considered as a single primary tumor. Bilateral tumors with two different histological types were considered primary unless otherwise stated. We defined MBC as BC with regional lymph nodes or distant organ metastasis. The occurrence of the MBC was considered an exposure factor, and the occurrence of the SPCs was considered the target event of our observation. The interval between the occurrence of the two primary cancers was considered the latency period of the SPCs. The exclusion criteria were as follows: 1) Patients diagnosed with SPCs within three months of BC; 2) Patients with only autopsy or death certificate records; 3) The status of the regional lymph nodes of BC patients and distant organs is unclear. Follow-up was conducted until the death of the patient, loss to follow-up, or until December 31, 2018.

## Statistical analysis

### Estimation of Standardized Incidence/Mortality Ratio

The relative incidence and mortality risk of SPCs in the general population were calculated using the SEER*Stat Multiple primary-standardized incidence ratios (MP-SIR) tool (version 8.3.8). The tool calculated the SIR by dividing the observed number of SPCs by the corresponding total person-years of follow-up and then multiplying by 10,000. The percentage contribution of BC and SPCs combination to the total SPCs incidence was calculated among all survivors, with a confidence interval (95% CI). The SIR (95% CI) was calculated as the ratio of the observed to the expected number of SPCs. The expected numbers of SPCs were calculated by a weighted sum of stratified incidence rates by latency from the reference population, which may include multiple primary cancers in a patient. The primary outcomes were incidence (per 10,000 person-years) and relative risk of developing SPCs among BC survivors (standardized incidence ratio [SIR]). The standardized mortality ratio (SMR) of SPCs was calculated using a similar approach. Statistically significant results were marked with * for ease of viewing.

### Kaplan-Meier method for survival analysis

The overall survival of the SPCs was analyzed by classifying the patients based on the status of BC metastasis, including NMBC (no regional lymph node metastasis, no distant organ metastasis); MBC-regional lymph node metastasis (regional lymph node metastasis, no distant organ metastasis); MBC-distant organ metastasis (distant organ metastasis). Cox regression analysis was performed to compare the risk of death in BC patients diagnosed with SPCs. For better representation of the results, the SPCs in MBC patients with a case number greater than 100 were analyzed.

### XGBoost model

XGBoost is a distributed gradient enhancement algorithm optimized based on CART and linear classifier. The principle of the XGBoost algorithm can be summarized as follows: feature vector with the corresponding (output) category yi:


, yiˆ=∑k=1Kfk(xi),fk∈F

Feature selection: univariate cox regression analysis was performed on the clinical characteristics retrieved from SEER database and statistically significant characteristics, including age at diagnosis, race, marital status, the incubation period of SPCs (month since index), stage, grade, distant site of metastasis and treatment information of two primaries, i.e., the site of SPCs and the hormone-receptor status of BC, were incorporated into the machine learning model to predict 3- and 5-year overall survival for SPCs. This analysis was performed before excluding patients who were alive but survived less than 3 or 5 years at the follow-up cut-off date. Before running the training program, a response variable was obtained for survival information, in which 1=survival and 0=death. One-hot encoding was performed for the three multi-classified variables like marital status, race, and the site of SPCs. Patients were randomly divided into training sets and test sets in the ratio of 7:3. The performance of SVM, decision tree (ID3), KNN, and XGBoost was compared using the training and test sets. Receiver operating characteristic (ROC) curve and area under the ROC curve (AUC) were used to evaluate the model.

## Results

The study included 17,077 female BC patients, of which 11,975 were NMBC patients and 5,102 were MBC patients, and the information was obtained from the database from 2010-2018. A total of 4537 cases of regional lymph node metastasis and 565 cases of distant organ metastasis in MBC patients were reported. Further classification of the patients with distant MBC revealed that 408 patients had bone metastasis; lung metastasis was reported in 173 patients, and 112 patients had liver metastasis (**Table 1** and **Table 2**). The number of cases of SPCs in patients with brain metastasis was only 23. On further classification based on the patient’s age, molecular subtype, race, etc., the number of cases in most categories was less than 5. The small number (<5) of observed cases were susceptible to confounding factors or occasionality (12). To avoid fallacious conclusions, BC patients with brain metastasis were not used for further analysis. The relevant results are presented in **Supplementary Table 1**.

**Table 1:** Standardized incidence ratios of SPCs in BC patients by characteristic.

characteristic	NMBC (N = 11975)				MBC (N = 5102)							
					regional node metastasis (N = 4537)				distant metastasis (N = 565)				
	Observe	Expected	SIR (O/E)	95%Cl	Observe	Expected	SIR (O/E)	95%Cl		Observe	Expected	SIR(O/E)	95%Cl	
Age														
<40	256	98.38	2.60*	2.29-2.94	176	72.26	2.44*	2.09-2.82		36	7.82	4.60*	3.24-6.38	
40-49	1123	846.93	1.33*	1.25-1.41	675	443.28	1.52*	1.41-1.64		77	32.38	2.38*	1.79-2.91	
50-59	2335	2223.68	1.05*	1.01-1.09	1131	964.7	1.17*	1.11-1.24		132	89.08	1.48*	1.24-1.76	
60-69	3844	4195.48	0.92*	0.89-0.95	1322	1387.52	0.95	0.90-1.01		157	145.14	1.08	0.92-1.27	
>=70	4417	4459.13	0.99	0.96-1.02	1233	1334.39	0.92*	0.87-0.98		163	172.73	0.94	0.81-1.10	
Subtype														
luminalA	8726	8735.56	1.00	0.98-1.02	3216	3012.49	1.07*	1.03-1.11		338	270.57	1.25*	1.12-1.39	
luminalB	867	934.82	0.93*	0.87-0.99	421	449.18	0.94	0.85-1.03		89	72.23	1.23	0.99-1.52	
Her2	351	369.95	0.95	0.85-1.05	200	181.38	1.10	0.96-1.27		39	30.05	1.3	0.92-1.77	
triple-negative	1310	1086.7	1.21*	1.14-1.27	468	354.66	1.32*	1.20-1.44		36	24.32	1.48*	1.04-2.05	
unknown	721	696.57	1.04	0.96-1.11	232	204.43	1.13	0.99-1.29		63	49.98	1.22	0.94-1.57	
Race														
white	9718	9984.03	0.97*	0.95-0.99	3562	3455.56	1.03	1.00-1.07		429	367.23	1.17*	1.06-1.28	
black	1259	1059.11	1.19*	1.12-1.26	587	479.06	1.23*	1.13-1.33		97	55.81	1.74*	1.41-2.12	
others	998	728.47	1.37*	1.29-1.46	388	253.59	1.53*	1.38-1.69		39	22.53	1.73*	1.23-2.37	
Grade														
well differentiated	3364	3392.16	0.99	0.96-1.03	603	621.15	0.97	0.89-1.05		40	36.47	1.1	0.78-1.49	
moderately differentiated	5022	5114.02	0.98	0.96-1.01	2056	1946.67	1.06*	1.01-1.10		233	169.48	1.37*	1.20-1.56	
poorly differentiated	3057	2809.97	1.09*	1.05-1.13	1654	1457.70	1.13*	1.08-1.19		178	136.29	1.31*	1.12-1.52	
unknown	532	507.45	1.05	0.96-1.14	224	176.62	1.27*	1.11-1.45		114	104.92	1.09	0.88-1.30	
Histologic type														
IDC	8826	8735.88	1.01	0.99-1.03	3288	3081.97	1.07*	1.03-1.10		359	294.55	1.22*	1.10-1.35	
ILC	1060	1117.29	0.95	0.89-1.01	547	492.46	1.11*	1.02-1.21		81	54.07	1.50*	1.19-1.86	
IDC and ILC(mixed)	645	647.87	1.00	0.92-1.08	332	309.36	1.07	0.96-1.20		28	21.57	1.30	0.86-1.87	
others	1444	1322.56	1.09*	1.04-1.15	370	318.36	1.16*	1.05-1.29		97	76.71	1.26*	1.01-1.52	
T stage														
T1	7731	7894.42	0.98	0.96-1.00	1479	1486.85	0.99	0.94-1.05		71	44.25	1.60*	1.22-1.98	
T2	2462	2263.48	1.09*	1.05-1.13	1744	1698.44	1.03	0.98-1.08		135	112.47	1.20*	1.01-1.42	
T3	301	247.10	1.22*	1.08-1.36	475	388.06	1.22*	1.12-1.34		79	57.25	1.38*	1.09-1.72	
T4	88	73.16	1.2	0.96-1.48	237	159.24	1.49*	1.30-1.69		115	100.59	1.14	0.95-1.37	
Tx	1393	1332.80	1.03	0.98-1.09	590	452.50	1.30*	1.20-1.41		165	132.53	1.25*	1.07-1.45	
N stage														
N0	/	/	/	/	/	/	/	/		110	90.12	1.22	0.99-1.45	
N1	/	/	/	/	2854	2853.02	1.00	0.96-1.04		203	159.87	1.27*	1.10-1.44	
N2	/	/	/	/	703	620.86	1.13*	1.05-1.22		44	41.71	1.05	0.76-1.42	
N3	/	/	/	/	428	312.98	1.37*	1.24-1.50		66	46.89	1.41*	1.09-1.76	
Nx	/	/	/	/	552	415.29	1.33*	1.22-1.44		142	108.48	1.31*	1.11-1.54	
Therapy														
without radiotherapy or chemotherapy	4017	3852.37	1.04*	1.01-1.08	839	795.31	1.05	0.98-1.13		159	140.98	1.13	0.96-1.33	
radiotherapy	5132	5193.57	0.99	0.96-1.02	752	719.09	1.05	0.97-1.12		79	65.73	1.20	0.95-1.48	
chemotherapy	1250	1207.95	1.03	0.98-1.09	980	931.43	1.05	0.99-1.12		184	155.66	1.18*	1.02-1.37	
radiotherapy and chemotherapy	1576	1569.70	1.00	0.96-1.05	1966	1756.32	1.12*	1.07-1.17		143	84.1	1.70*	1.44-2.00	

*p<0.05 compared to the general population.

**Table 2:** Standardized incidence ratios of SPCs in distant MBC patients by characteristics.

	Bone metastasis (N = 408)				Lung metastasis (N = 173)				Liver metastasis (N = 112)			
	Observe	Expected	SIR (O/E)	95%Cl	Observe	Expected	SIR (O/E)	95%Cl	Observe	Expected	SIR (O/E)	95%Cl
Age												
<40	26	5.74	4.53*	2.96-6.64	9	1.57	5.74*	2.63-10.90	7	2.61	2.69*	1.08-5.54
40-49	56	24.48	2.29*	1.73-2.97	29	7.75	3.74*	2.51-5.38	16	8.78	1.82*	1.04-2.96
50-59	99	68.34	1.45*	1.18-1.76	36	24.70	1.46*	1.02-2.02	37	23.15	1.60*	1.13-2.20
60-69	112	111.16	1.01	0.83-1.21	41	45.78	0.90	0.64-1.21	28	26.89	1.04	0.69-1.50
>=70	115	128.37	0.90	0.74-1.08	58	57.20	1.01	0.77-1.31	24	24.52	0.98	0.63-1.46
Subtype												
Luminal A	256	221.26	1.20*	1.06-1.35	101	76.90	1.31*	1.07-1.60	50	36.38	1.37*	1.02-1.81
luminal B	68	50.21	1.35*	1.05-1.72	19	24.09	0.79	0.47-1.23	27	22.46	1.20	0.79-2.75
Her2	19	15.94	1.19	0.72-1.86	14	10.35	1.35	0.74-2.27	18	13.61	1.32	0.78-2.09
triple-negative	23	12.58	1.83*	1.16-2.74	12	11.22	1.07	0.55-1.87	7	5.74	1.22	0.49-2.51
unknown	42	48.36	0.87	0.60-1.22	27	14.42	1.87*	1.23-2.72	10	7.75	1.29	0.62-2.37
Race												
white	318	281.08	1.13*	1.01-1.26	124	108.77	1.14	0.95-1.36	81	68.94	1.17	0.93-1.46
black	59	39.60	1.49*	1.13-1.92	40	19.17	2.09*	1.49-2.84	17	11.50	1.48	0.86-2.37
others	31	16.53	1.88*	1.27-2.66	9	8.29	1.09	0.50-2.06	14	5.22	2.68*	1.47-4.50
Grade												
well differentiated	28	30.92	0.91	0.60-1.31	11	8.41	1.31	0.65-2.34	7	4.65	1.50	0.60-3.10
moderately differentiated	177	135.40	1.31*	1.12-1.51	70	49.40	1.42*	1.10-1.79	40	27.81	1.44*	1.03-1.96
poorly differentiated	121	88.45	1.37*	1.14-1.63	52	49.25	1.06	0.79-1.38	48	35.25	1.36*	1.00-1.81
unknown	82	83.32	0.98	0.78-1.22	40	29.92	1.34	0.96-1.82	17	18.22	0.93	0.54-1.49
Histologic type												
IDC	255	212.71	1.20*	1.06-1.36	122	102.00	1.20	0.99-1.43	69	61.98	1.11	0.87-1.41
ILC	72	49.52	1.45*	1.14-1.83	11	5.02	2.19*	1.09-3.92	13	6.94	1.87	1.00-3.20
IDC and ILC(mixed)	22	18.71	1.18	0.74-1.78	3	4.12	0.73	0.15-2.13	5	3.05	1.64	0.53-3.83
others	59	57.16	1.03	0.79-1.33	37	25.85	1.43*	1.01-1.97	25	13.97	1.79*	1.16-2.64
T stage												
T1	47	34.63	1.36	1.00-1.80	18	9.73	1.85*	1.10-2.92	15	8.40	1.79	1.00-2.95
T2	94	84.94	1.11	0.89-1.35	37	29.86	1.24	0.87-1.71	29	21.06	1.38	0.92-1.98
T3	63	42.44	1.48*	1.14-1.90	20	16.54	1.21	0.74-1.87	10	10.67	0.94	0.45-1.72
T4	79	74.07	1.07	0.84-1.33	45	41.89	1.07	0.78-1.44	30	20.26	1.48	1.00-2.11
Tx	125	102.02	1.23*	1.02-1.46	53	38.97	1.36*	1.02-1.78	28	25.55	1.10	0.73-1.58
N stage												
N0	71	69.28	1.02	0.80-1.29	41	22.68	1.81*	1.30-2.45	19	14.98	1.27	0.76-1.98
N1	141	119.27	1.18	1.00-1.39	57	52.10	1.10	0.83-1.42	45	32.85	1.37	1.00-1.83
N2	31	29.99	1.03	0.70-1.47	14	13.75	1.02	0.56-1.71	12	7.80	1.54	0.79-2.69
N3	56	36.05	1.55*	1.17-2.02	16	13.86	1.15	0.66-1.87	12	8.77	1.37	0.71-2.39
Nx	109	83.49	1.31*	1.07-1.57	45	34.69	1.30	0.95-1.74	24	21.54	1.11	0.71-1.66
Therapy												
without radiation or chemotherapy	112	105.11	1.07	0.88-1.28	54	44.71	1.21	0.91-1.58	21	18.81	1.12	0.69-1.71
radiation	68	60.22	1.13	0.88-1.43	20	13.78	1.45	0.89-2.24	9	4.74	1.90	0.87-3.61
chemotherapy	112	107.5	1.04	0.86-1.25	58	57.14	1.02	0.77-1.31	57	44.83	1.27	0.96-1.65
radiation and chemotherapy	116	65.27	1.78*	1.47-2.13	41	21.37	1.92*	1.38-2.60	25	17.57	1.42	0.92-2.10

*p<0.05 compared to the general population.

### The patterns of SPCs in BC patients with different metastasis

**Figure 2:**
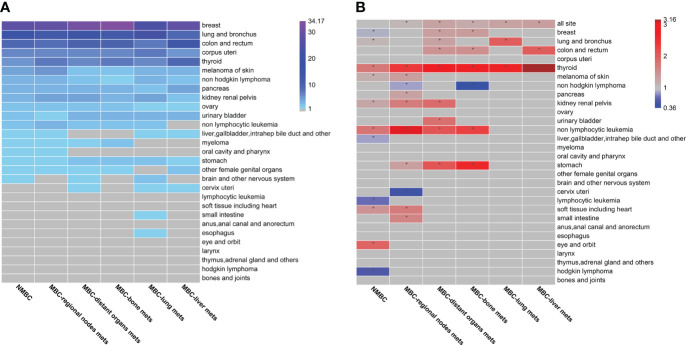
The patterns of second primary cancers (SPCs) in Breast cancer (BC) patients with different metastasis status **(A)** Calculated by dividing the number of SPC cases observed per unit by the total number of SPCs observed. The grey cells indicate tumor types that are less than 1% of the total number of SPCs. Percentage values are shown in **Supplementary Table 2**. **(B)** Standard incidence rates (SIRs) are calculated as the ratio of the number of SPCs observed to the number of SPCs expected in the general population. Cells marked with an * symbol show a statistically significant association between first primary breast cancer (BC) and SPCs. Blue cells indicate SIRs that are statistically significantly lower than expected according to a qualifying statistical test (number of SPCs observed ≥ 5). Red cells indicate SIRs that are statistically significantly higher than expected according to a qualifying statistical test (number of SPCs observed ≥ 5). Gray cells indicate SIRs, which are not statistically significant or associations not tested due to the small number of SPCs observed. Point estimates and 95% CIs are shown in **Supplementary Table 3**.

The incidence of SPCs in BC patients with different metastasis was investigated. The results reveal 29 types of SPCs (**Figure 2A** and **Supplementary Table 2**). The top ten SPCs in NMBC patients were breast (27.47%), lung and bronchus (15.22%), colon/rectum (8.79%), corpus uteri (6.76%), thyroid (4.98%), melanoma of skin (4.65%), non-Hodgkin’s lymphoma (3.85%), pancreas (3.63%), kidney/renal pelvis (3.5%), and ovarian (2.62%). These accounted for over 80% of the SPCs in BC patients. The top five SPCs types reported were nearly the same in both NMBC and MBC patients, except for thyroid cancers, the fourth most common SPCs reported in MBC patients. The ranking of melanoma of skin, non-Hodgkin’s lymphoma, and ovarian cancer decreased with the progression of metastasis, whereas the kidney renal pelvis cancer ranking increased with metastasis development. These results indicate that metastasis might affect the incidence of SPCs in BC patients.

### Increase in SIR of SPCs in most MBC patients

The role of metastasis on the SIR of SPCs in BC patients was explored (**Figure 2B** and **Supplementary Table 3**). The results reveal that the SIR (1.01, 95% CI, 0.99–1.03, p>0.05) of SPCs in NMBC patients was similar to the general population. Patients with regional lymph node metastasis had an 8% increased risk of SPCs (SIR=1.08, 95% CI, 1.05–1.11, p<0.05), and patients with distant metastasis had a 26% increased risk of SPCs (SIR=1.26, 95% CI, 1.16–1.37, p<0.05). These results show that the SIR of SPCs in BC patients increased with the progression of metastasis. In NMBC patients, the SIR of seven SPCs, such as eye/orbit, non-lymphocytic leukemia, thyroid, soft tissue including heart, kidney/renal pelvis, melanoma of skin, and lung/bronchus cancers, were higher than the general population. Compared to NMBC patients, a decrease in SIR of the eye/orbit and an increase in the SIR of the other six SPCs were observed in regional nodes or distant MBC patients. To our surprise, a decrease in the risk of second primary BC (SIR=0.90, 95% CI, 0.86–0.93, p<0.05) was reported for NMBC patients. Similar results were observed for the other three types of SPCs (liver, gallbladder, intrahepatic bile duct, and other; lymphocytic leukemia; Hodgkin lymphoma). However, the SIR of all four SPCs was higher in MBC patients. In NMBC patients, the SIR of 18 SPCs was similar to the general population. Further, an increased risk of five SPCs (stomach, urinary bladder, colon and rectum, small intestine, pancreas) was observed in MBC patients, whereas a decrease in the SIR of two SPCs (non-Hodgkin lymphoma, cervix uteri) was reported in regional nodes MBC patients.

For MBC patients with distant metastasis, the risk of SPCs of the thyroid was higher in MBC patients with bone, lung, and liver metastasis, while the risk of SPCs of lung and bronchus cancer (SIR=2.02, 95% CI, 1.43–2.78, p<0.05) was only higher in MBC patients with lung metastasis. Most high-risk SPCs, such as stomach, colon, rectum, thyroid, non-lymphocytic leukemia, and breast, were observed in patients with bone metastasis. However, the SIR (0.36; 95% CI, 0.12–0.87, p<0.05) of second primary non-Hodgkin lymphoma significantly decreased in bone MBC patients. Overall, the MBC patients had a higher risk of developing most SPCs.

### Association between SIR and general characteristics of SPCs in BC patients with different metastatic status

In order to understand the relationship between metastasis and SPCs, the association between SIR and general characteristics of SPCs in BC patients with different metastatic status was investigated (**Table 1**). The SIR of SPCs in all patients below the age of 40 was the highest, which decreased with age. The risk of developing SPCs in whites was lower than in blacks and other races. In general, patients with poorly differentiated cancer cells, large tumor size, and late N stage had an increased risk of SPCs. However, in distant MBC patients, an increased SIR of SPCs was reported even at the earlier T1(SIR=1.60, 95% CI, 1.22–1.98, p<0.05) and N1 (SIR=1.27, 95% CI, 1.10–1.44, p<0.05) stage. For pathological types, only in IDC and ILC (mixed) types no elevation in SIR was observed with the progression of metastasis. Meanwhile, a significantly increased risk of SPCs was observed in MBC patients treated with both radiotherapy and chemotherapy. The BC patients were then classified based on the molecular subtypes, and the results reveal that patients with triple-negative BC had an increase in the SIR of SPCs, and in the luminal A subtype, an increase in SIR of SPCs was observed with metastasis progression. However, in patients with Her-2 positive and luminal B subtype, the SIR of SPCs remained unchanged.

The SIR and general characteristics of SPCs in the bone, lung, and liver MBC patients were further analyzed (**Table 2**). For those patients younger than 50 years, the risk of SPCs in patients with lung metastasis was higher compared to patients with bone or liver metastases. In any race, the patients with bone MBC had an elevated risk of SPCs; however, the SPCs risk increased in black women with lung MBC and women with liver MBC of other races. In pathological types like IDC, an increase in the SIR of SPCs was observed in patients with bone metastasis; however, in ILC type, an increase in the SIR of SPCs was observed in patients with bone or lung metastasis. Further, in other pathological types, the SIR of SPCs was elevated in patients with lung or liver metastasis. Patients with bone MBC have an increased risk of SPCs at a late stage (T3: SIR=1.48, 95% CI, 1.14–1.90, p<0.05; N3: SIR=1.55, 95% CI, 1.17–2.02, p<0.05). On the contrary, an increased risk of SIR was observed in patients with lung MBC at an early stage (T1: SIR=1.85, 95% CI, 1.10–2.92, p<0.05; N0: SIR=1.81, 95% CI 1.30–2.45, p<0.05). In the luminal A subtype, all the patients with distant metastases were at increased risk of SPCs; however, in patients with triple-negative BC and luminal B subtypes, an elevated risk of SPCs was only observed in patients with bone metastasis. There was no statistical difference in the SPCs risk in the Her-2 subtype.

**Figure 3:**
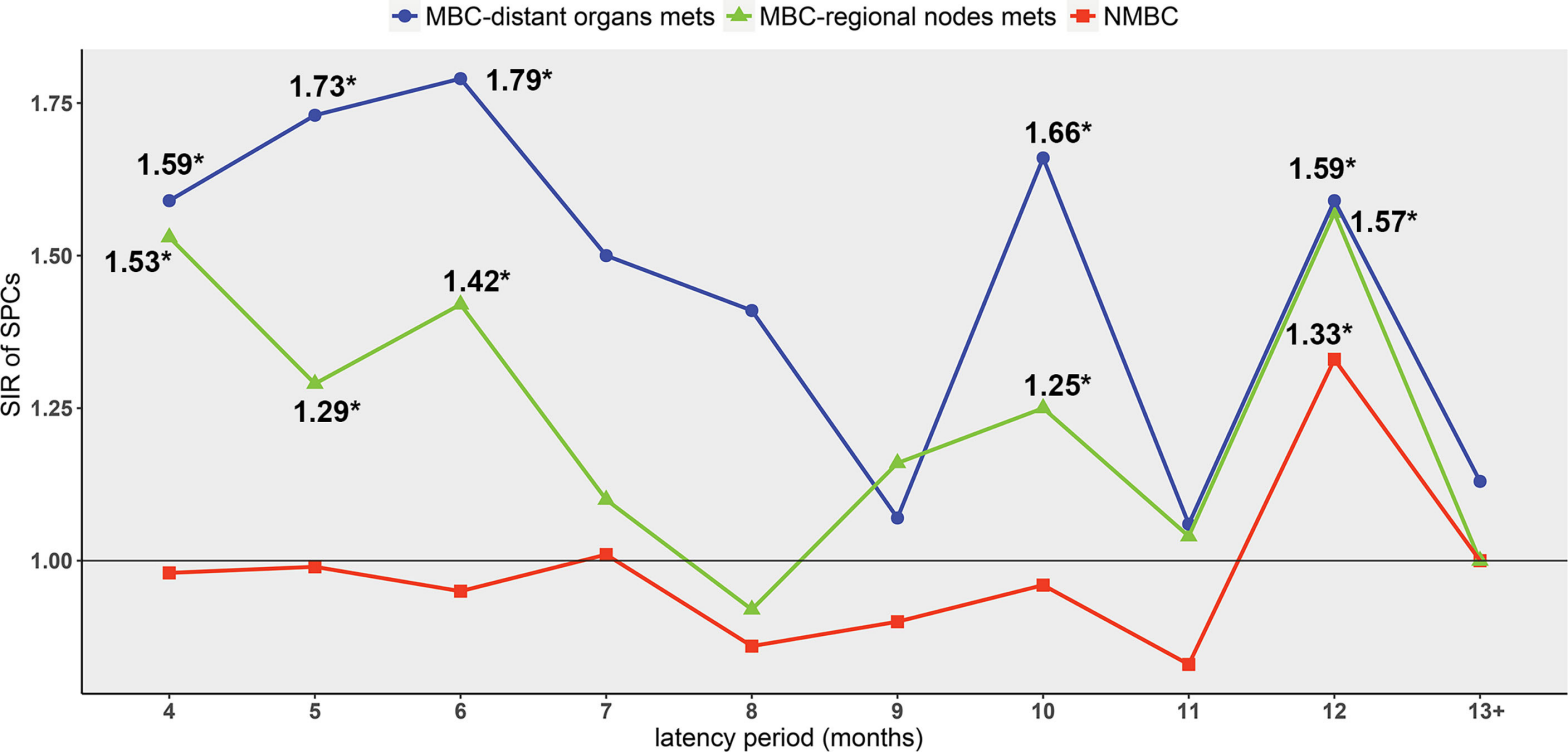
The SIR of SPCs varies with the latency period *p < 0.05 compared to the general population. Point estimates and 95% CIs are shown in **Supplementary Table 4**.

Further, the relationship between the risk of SPCs and the time elapsed after BC diagnosis was explored. The peak of SPCs risk occurred earlier in MBC patients (4-6 months and 10 months) compared to NMBC patients (12 months). At 12 months, the risks of SPCs were significantly elevated in all the three groups (NMBC: SIR=1.33, 95% CI 1.18–1.50, p<0.05; regional nodes MBC: SIR=1.57, 95% CI 1.30–1.87, p<0.05; distant MBC: SIR=1.59, 95% CI 1.01–2.49, p<0.05) (**Figure 3**, **Supplementary Table 4**). After 12 months, there was no significant increase in SIR of SPCs risk in all groups (**Supplementary Table 4**).

### Increase in SMR of SPCs in MBC patients

**Figure 4:**
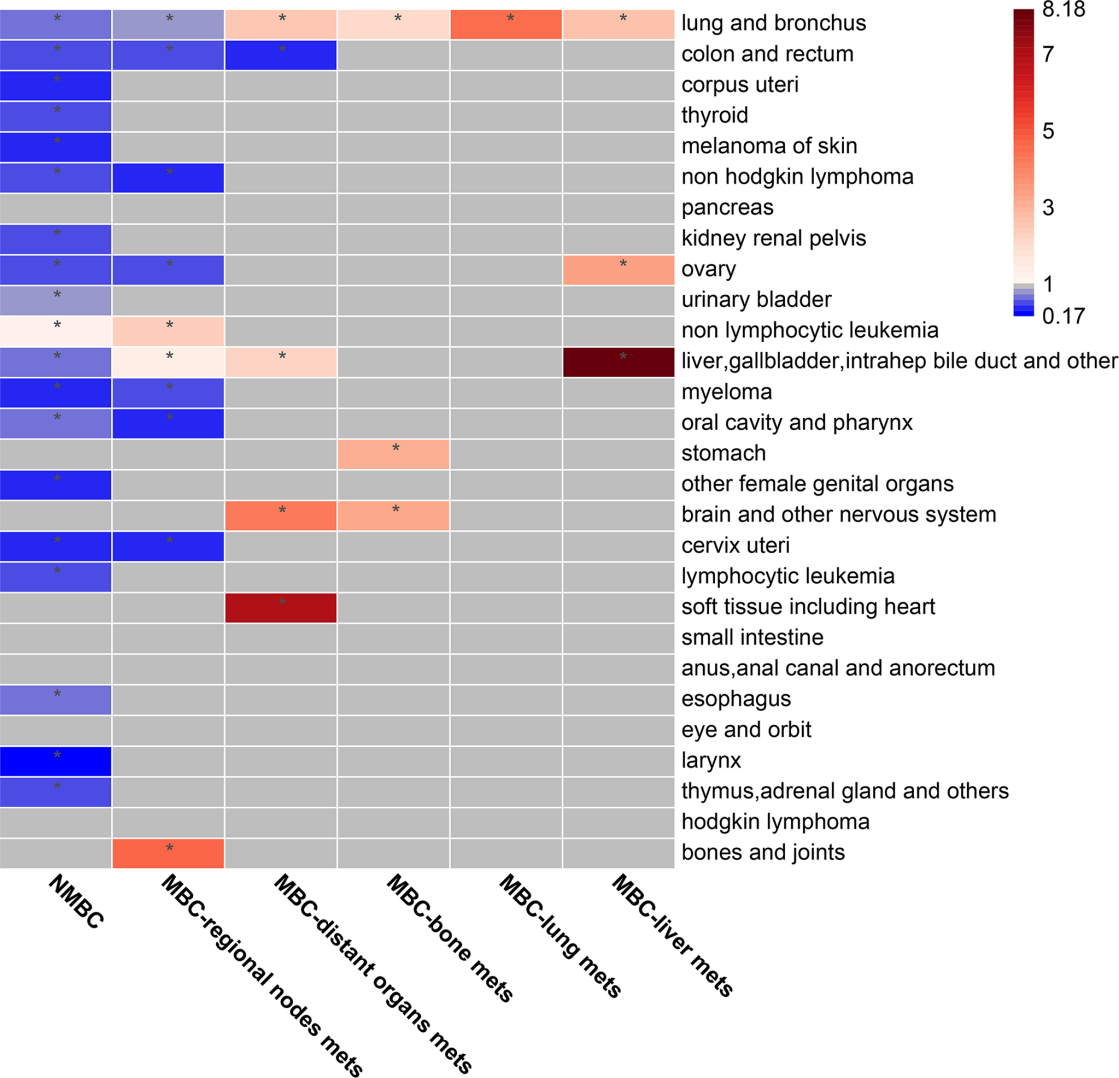
The standard mortality rate (SMR) of SPCs in BC patients with different metastasis status. The SMR is calculated as the ratio of observed SPC deaths to the expected number of cancer deaths in the general population. The cells marked with an * symbol indicate statistically significant association between first primary breast cancer (BC) and SPCs. Blue cells indicate SMRs that are statistically significantly lower than expected according to a qualifying statistical test (number of SPCs observed ≥ 5). Red cells indicate SMRs that are statistically significantly higher than expected according to a qualifying statistical test (number of SPCs observed ≥ 5). Grey cells indicate SMRs that are not statistically significant, or no association was tested due to the small number of SPCs observed. Point estimates and 95% CIs are shown in **Supplementary Table 5**.

No elevation in the SMR of the SPCs was observed in NMBC patients compared to the general population, except for non-lymphocytic leukemia (SMR=1.25, 95% CI, 1.01–1.52, p<0.05). Nevertheless, the SMR was reduced in most of SPCs **(** **Figure 4**, **Supplementary Table 5**). Further, it was evident that the SMR of the SPCs increased with the progression of metastasis, especially in the second primary liver/gallbladder, soft tissue, bone/joints, brain/other nervous system, and lung/bronchus cancers. Notably, in patients with bone or liver metastases, a significant increase in the SMR of the second primary stomach (SMR=3.16, 95% CI, 1.16–6.88, p<0.05) and ovarian (SMR=3.43, 95% CI, 1.11–8.01, p<0.05) cancers, was observed.

### Prognosis after developing SPCs in BC patients with different metastatic status

**Figure 5:**
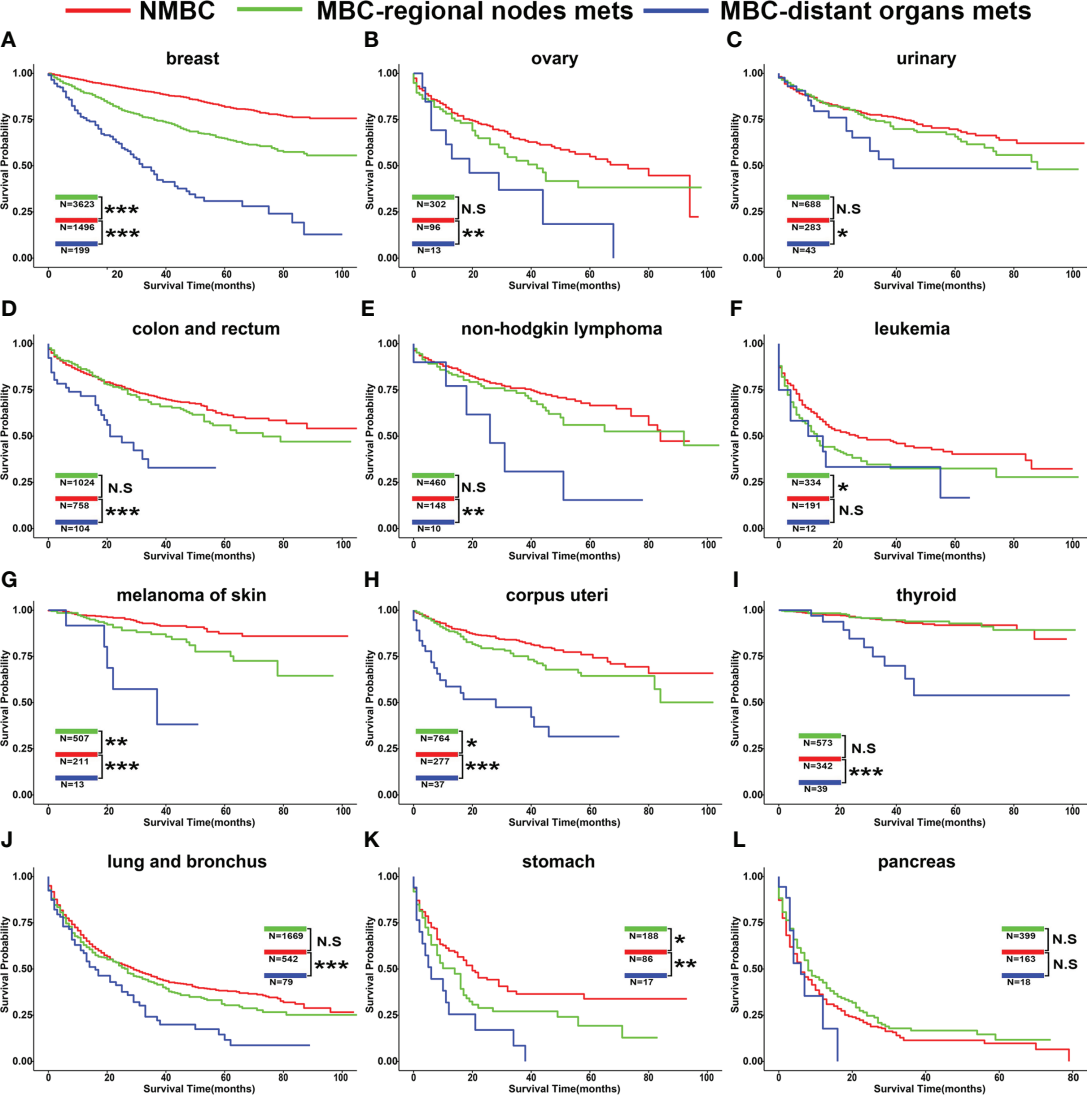
The overall survival of SPCs classified by the metastatic status of breast cancer. Site of SPCs: **(A)** breast, **(B)** ovary, **(C)** urinary, **(D)** colon and rectum, **(E)** non-Hodgkin lymphoma, **(F)** leukemia, **(G)** melanoma of skin, **(H)** corpus uteri, **(I)** thyroid, **(J)** lung and bronchus, **(K)** stomach, **(L)**. pancreas. HR and 95% CIs are shown in **Supplementary Table 6**.*p < 0.05; **p < 0.01; ***p < 0.001. N.S, not significant.

After BC patients were diagnosed with SPCs, whether the patient prognosis was affected by metastatic status in different types of SPCs was unclear. Hence, we evaluated the overall survival (OS) of these patients (**Figure 5**, **Supplementary Table 6**). For most BC patients with SPCs, the OS of patients with distant metastasis was significantly poor compared to patients with regional lymph node metastasis and NMBC patients. However, no difference was observed between patients with second primary leukemia and pancreas cancer (**Figures 5F, L**). For most BC patients with regional lymph node metastasis, the OS was similar to NMBC patients. However, if BC patients with regional lymph node metastasis were diagnosed with SPCs, such as breast, leukemia, melanoma of skin, corpus uteri, and stomach cancer, the prognosis of the patients was poor compared to NMBC patients **(Figures 5A, F, G, H, K**).

### Establishment of predictive models for estimating prognosis in MBC patients with SPCs

**Figure 6:**
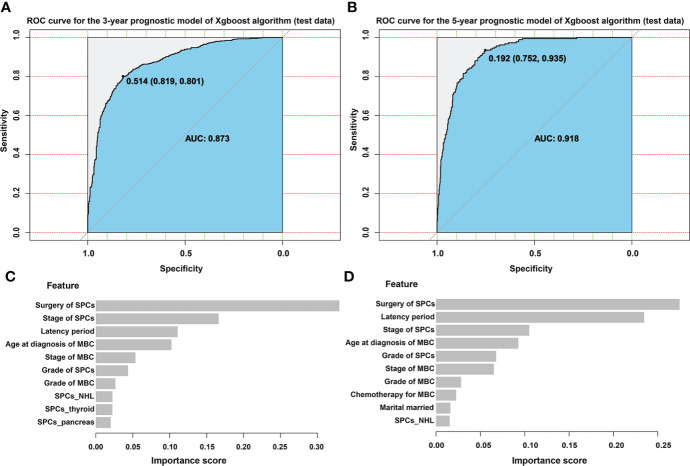
XGBoost model evaluationROC: receiver operating characteristic curve; AUC: area under the curve **(A)** ROC curve for the 3-year prognostic model of XGBoost algorithm (test data), **(B)** ROC curve for the 5-year prognostic model of XGBoost algorithm (test data), **(C)** The ranking of clinical characteristics in terms of importance in the 3-year prognostic model. The x-axis of the graph is the feature importance score. The closer the score is to 1, the more important the feature in predicting the prognosis of the patient in the model. **(D)** The ranking of clinical characteristics in terms of importance in the 5-year prognostic model. The x-axis of the graph is the feature importance score. The closer the score is to 1, the more important the feature in predicting the prognosis of the patient in the model.

Based on the above results, predictive models were created to estimate 3-year and 5- year prognosis in MBC patients after diagnosis with SPCs. Univariate Cox regression analysis was first performed (**Supplementary Table 7**) on the model. The model was then tested and adjusted repeatedly, and the available parameters were identified (Supplementary R code). The ranking of clinical characteristics based on their importance in the model was assessed. The results showed that surgery, stage of SPCs, latency period, and age at which MBC was diagnosed were the top four determinants of patient survival (**Figures 6C, D**). Of these characteristics, the surgery in SPCs patients was the most important factor. The ROC curves of the predictions for the training and the test set were constructed, and the corresponding AUC was calculated. The XGBoost model created by us had an excellent ability to predict the 3-year and 5-year survival of SPCs in MBC patients (3-year survival in test set: AUC=0.873; 5-year test set: AUC=0.918; **Figures 6A, B**), compared to SVM (3-year survival in test set: AUC=0.689; 5-year survival in test set: AUC=0.729), ID3 (3-year survival in test set: AUC=0.763; 5-year survival in test set: AUC=0.782), and KNN model (3-year survival in test set: AUC=0.712; 5-year survival in test set: AUC=0.798; **Table 3**).

**Table 3:** Performance of prognostic models built by machine learning algorithms on test sets.

	AUC	
	3-year survival	5-year survival
XGBoost	0.873	0.918
ID3	0.763	0.782
SVM	0.689	0.729
KNN	0.712	0.798

## Discussion

In this study, we investigated the patterns of SPCs in BC patients with different status of metastasis and identified the most common SPCs of BC patients. Our analysis revealed that metastasis affects the patterns of SPCs and increases the SIR and SMR of SPCs in BC patients. Interestingly, the effect of metastasis on prognosis in SPCs patients was dependent on the type of SPCs. Further, we created a XGBoost model to predict the 3-year and 5-year survival of MBC patients diagnosed with SPCs. Our results reveal that the risk of second malignancies in BC patients was associated with the patient’s age, race, T/N staging, molecular subtype, etc. However, in patients with different metastasis status, the characteristics of the SPCs risk may be varied. In MBC patients, the peak of SPCs risk occurred earlier compared to NMBC patients. Interestingly, after 12 months of BC diagnosis, the risks of SPCs in all patients were significantly elevated, which shows the importance of early diagnosis of SPCs, especially in the first year.

Our results show that the most common sites of SPCs were breast, lung/bronchus, colon/rectum, corpus uteri, and thyroid, consistent with the previous study based on SEER population (17). Further, a study by Qian et al. (17) lacked data on metastasis and comparison between NMBC and MBC patients. In comparison, our results revealed the top ten SPCs in BC patients. The top five types of SPCs were nearly the same in both NMBC and MBC patients, except thyroid cancers, which ranked fourth in MBC patients. The ranking of melanoma of skin, non-Hodgkin’s lymphoma, and ovarian decreased with the progression of metastasis, while the ranking of kidney renal pelvis cancers increased with metastasis development. The frequency of kidney renal pelvis cancer was low after a first primary BC diagnosis, especially in MBC patients. These novel findings suggest that metastasis may affect the incidence of SPCs.

To further verify the above hypothesis, the analysis revealed that compared to the general population, no increased risk of SPCs was observed in NMBC patients, which account for the most BC survivors. The risk of second primary BC was even lower in NMBC patients. However, our results contradict previous studies which reported an increased risk of SPCs in NMBC patients (17, 18). It is important to note that previous studies failed to incorporate the peculiarities of regional lymph nodes MBC patients and were classified in the same “non-metastatic” category. In our study, regional lymph nodes MBC patients had a mildly increased risk of SPCs. To the best of our knowledge, for the first time, our results reveal a significantly higher risk of SPCs in BC patients with distant metastasis. It is widely accepted that the risk of SPCs increases in patients who survive longer (12); however, our analysis shows contradictory results to the widely accepted phenomenon. The SIR of SPCs increased with the progression of metastasis. Compared to NMBC, patients with distant organ metastasis had a higher risk of SPCs, such as breast, thyroid, stomach, kidney/renal, urinary bladder, lung/bronchus, colon/rectum cancers, and non-lymphocytic leukemia. A previous study on metastatic melanoma showed an increased risk of the second primary intestine, lung/bronchus, kidney cancers, and myeloma (19). Another report suggests that metastatic cancer survivors of uncertain primary origin were at increased risk of developing occult gastrointestinal tumors (20). To the best of our knowledge, apart from our study, only Deng et al. (19) and Hannouf et al. (20) have explored the risk of SPCs in metastatic cancers. However, these studies fail to show the results in light of comparison with non-metastatic cancer patients. Hence, these results imply that attention should be paid to the risk of developing SPCs in metastatic cancer patients.

Interestingly, our results show a decrease in SMR of most SPCs in NMBC patients, whereas an increase was observed with the progression of BC metastasis. Usually, the prognosis of NMBC patients is better due to frequent periodic physical examinations of the patients. This leads to early diagnosis and treatment of SPCs and could contribute to the low SMR observed in most SPCs. The results also reveal that SMR and SIR of the second primary stomach were significantly higher in BC patients with bone metastasis since 75% of patients with metastatic BC have bone metastasis (7). Further, we suggest that the screening of gastric cancer for distant MBC patients should be increased. The SMR and SIR of second primary lung/bronchus cancer were significantly higher in BC patients with lung metastasis. This indicates that in the case of distant MBC patients, analysis of lung lesions as primary or metastasis is essential; hence, SPCs should be considered. A significantly higher risk of second primary thyroid cancer was also observed in our study, which is consistent with previous studies (21–23). Moreover, our results show a gradient in risk with metastatic modality (SIR: NMBC: 1.72 vs. MBC-regional node: 2.35 vs. MBC-distant organ: 2.77), and the prognosis of distant MBC was poor after diagnosis with second primary thyroid cancer.

Consistent with previous studies, our results show that younger patients were more likely to develop SPCs compared to older patients (24, 25). Further, the patients older than 60 years did not show an increased risk of SPCs regardless of metastasis status. A previous study in Korea reported that the most common SPCs of BC were thyroid, stomach, and corpus cancers (26), which differ from our results. This indicates that differences in race may affect the risk of SPCs (27). In our study, we observed a lowered risk of developing SPCs in the whites compared to the blacks and other races. However, patients with bone MBC of any race had an elevated risk of SPCs, but the risk was only increased in black women with lung MBC and women with liver MBC of other races. We also report that the risk of SPCs in BC patients with regional lymph node metastasis increases with T and N staging. However, the BC patients with distant metastasis showed a significantly high risk of SPCs at the T1N1 stage, contrary to results in patients with regional lymph node metastasis. Hence, additional studies are required to understand the discrepancies in the results. Regarding BC molecular subtypes, our analysis shows that in triple-negative BC patients, an increase in the SIR of SPCs was observed in all patients. In the luminal A subtype, the SIR of SPCs was elevated with metastasis development. However, the SIR of SPCs in Her-2 positive and luminal B subtype remained unchanged, which is partially consistent with previous studies (28, 29). Further, all the patients with distant metastases were at increased risk of SPCs in the luminal A subtype; however, in triple-negative and luminal B subtypes, this elevated risk of SPCs was only observed in bone metastatic patients. These results suggest that additional attention should be given to BC patients with bone metastases. Together, these results show that metastasis plays an important role in developing SPCs.

To the best of our knowledge, our study is the first of its kind to evaluate the prognosis of BC patients diagnosed with SPCs with different metastasis status. Our results show metastasis had a smaller effect on the overall prognosis of SPCs, such as liver and pancreas cancers with poor prognosis, while for those SPCs with good prognosis, for instance, thyroid cancer, corpus uteri carcinoma, and skin melanoma (mostly at an early stage), BC metastasis was the main reason for the death. However, it is difficult to estimate the prognosis of patients after diagnosis of SPCs based on these results. Previous studies have established columnar plots to predict the probability of developing SPCs and BC-specific survival. However, the accuracy of previous models was low; hence, high-precision models are required for prediction. To the best of our knowledge, no previous studies have demonstrated a model estimating the prognosis in MBC patients with SPCs. Therefore, in this study, we built an XGBoost model based on the clinical characteristics of SPCs patients. The results show that our model could accurately predict the prognosis of SPCs patients compared to previous traditional machine learning methods. Among the clinical characteristics, the surgical history, stage and latency period of SPCs, and age at which MBC was diagnosed were the four most critical factors in the prognosis of SPCs. These results suggest that SPCs may be the main cause of death after BC (30–32). Furthermore, we demonstrated that stage, grade, and chemotherapy in MBC had higher scores in the model characteristics, which showed that MBC influences the OS of patients with SPCs.

Furthermore, the results show that patients with MBC developed SPCs earlier than NMBC patients, specifically in the first year after diagnosis with BC. This will aid in classifying the patients based on risk for SPCs in BC survivors and will provide the basis for designing their screening and follow-up strategies. Although the analysis was conducted on data obtained from a large and accurate cancer database, the association at genetic levels is still lacking. Genetic change is an important endogenous cause of metastasis and SPCs, and the genotype-phenotype correlation of SPCs remains unclear. Current studies on SPCs genotypes are limited, and additional research is required to understand the relationship between SPCs and metastasis at the genetic level. Despite these limitations, our study still had important implications for BC survivors.

In conclusion, we investigated the relationship between metastasis and SPCs in women with BC. We compared the SIR, SMR, and characteristics of SPCs in BC patients with different metastasis status and constructed an XGBoost model for evaluating prognosis in MBC patients with SPCs. Our results demonstrated an important role of metastasis in developing SPCs and provide a more theoretical basis for clinical follow-up and screening of SPCs.

## Data availability statement

The original contributions presented in the study are included in the article/**Supplementary Material**. Further inquiries can be directed to the corresponding authors.

## Author contributions

CL and JQ conceived and designed the study and completed the article. CL, ML, JL, YW, XC, WW, and SS conducted the statistical analysis and explained the results of this study. CF, FW, CD, and YC were responsible for creating figures and tables for this study. SZ, YZ, and XZ scrutinized the whole process of this study and critically reviewed the initial draft of the manuscript. All authors contributed to the article and approved the submitted version.

## Funding

This work was funded in part by the following: National Science Foundation of China (81903856, to XZ; 82174164, to SZ, 82103569, to JQ); Key Science and Technology Program of Shaanxi Province (2021KW-57, to XZ; 2021KW-60, to JQ). Scientific research fund of the Second Affiliated Hospital of Xi’an Jiaotong University (RC(XM)202004, to XZ). Free exploring fund of Xi’an Jiaotong University (xzy012022096, to XZ; xzy012022097 to JQ). Medical "basic - clinical" integration and innovation project of Xi 'an Jiaotong University (YXJLRH2022088 to JQ).

## Acknowledgments

We would like to thank all the developers of the R programming package for selflessly sharing their code.

## Conflict of interest

The authors declare that the research was conducted in the absence of any commercial or financial relationships that could be construed as a potential conflict of interest.

## Publisher’s note

All claims expressed in this article are solely those of the authors and do not necessarily represent those of their affiliated organizations, or those of the publisher, the editors and the reviewers. Any product that may be evaluated in this article, or claim that may be made by its manufacturer, is not guaranteed or endorsed by the publisher.
