# Supplementary material for: Relationship between metastasis and second primary cancers in women with breast cancer
Source: Front Oncol. 2022 Sep 29;12:942320. doi: 10.3389/fonc.2022.942320 (PMC9556865; doi:10.3389/fonc.2022.942320)
Supplement: Supplementary file 8 [file DataSheet_1.docx]

**R code of Machine learning algorithm model**

**Xgboost**

**setwd(dir="c:/Users/a/Desktop/")**

**library("caret")**

**library("xgboost")**

**library("stringr")**

**library("Matrix")**

**library("pROC")**

**library("DiagrammeR")**

**#** **The data set was divided into training set and test set in a ratio of 7:3**

**seer<-read.csv("xgboost3.csv",header = TRUE)**

**set.seed(70)**

**train_sub <- sample(nrow(seer),7/10*nrow(seer))**

**train_data <- seer[train_sub,]**

**test_data <- seer[-train_sub,]**

**#** **Training set data preprocessing**

**#** **Transform the independent variable into a matrix**

**traindata1 <- data.matrix(train_data[,c(1:44)])**

**#** **The matrix is transformed into sparse matrix by matrix function**

**traindata2 <- Matrix(traindata1,sparse = TRUE)**

**traindata3 <- train_data[,45]**

**#** **Concatenate independent and dependent variables into a list**

**traindata4 <- list(data=traindata2,label=traindata3)**

**#** **Build the xgb.DMatrix object**

**dtrain <- xgb.DMatrix(data = traindata4$data,label=traindata4$label)**

**# Training set data preprocessing**

**# Transform the independent variable into a matrix**

**testset1 <- data.matrix(test_data[,c(1:44)])**

**# The matrix is transformed into sparse matrix by matrix function**

**testset2 <- Matrix(testset1,sparse = TRUE)**

**testset3 <- test_data[,45]**

**# Concatenate independent and dependent variables into a list**

**testset4 <- list(data=testset2,label=testset3)**

**# Build the xgb.DMatrix object**

**dtest <- xgb.DMatrix(data = testset4$data,label=testset4$label)**

**#** **Training model and Adjust the parameters**

**xgb<-xgboost(data=dtrain,max_depth=7,eta=0.17,subsample=0.7,objective='binary:logistic',nround=25)**

**#** **feature importance**

**importance <- xgb.importance(traindata2@Dimnames[[2]],model = xgb)**

**xgb.plot.importance(importance_matrix = importance)**

**#** **Predict on the test set**

**pred1 <- data.frame(predict(xgb,testset2,type='response'))**

**xgb_lr.train.modelroc<-roc(test_data$Group,pred1$predict.xgb..testset2..type....response..)**

**plot(xgb_lr.train.modelroc,print.auc=TRUE,auc.polygon=TRUE,grid=c(0.1,0.2),grid.col=c("green","red"),max.auc.polygon=TRUE,auc.polygon.col="skyblue",print.thres=TRUE,main='ROC curve of Xgboost algorithm')**

**# Predict on the train set**

**pred2<- data.frame(predict(xgb,traindata2,type='response'))**

**xgb_lr.train.modelroc<-roc(train_data$Group,pred2$predict.xgb..traindata2..type....response..)**

**plot(xgb_lr.train.modelroc,print.auc=TRUE,auc.polygon=TRUE,grid=c(0.1,0.2),grid.col=c("green","red"),max.auc.polygon=TRUE,auc.polygon.col="skyblue",print.thres=TRUE,main='ROC curve of Xgboost algorithm')**

**xgb.plot.tree(model = xgb, trees =0, plot_width = 1000,plot_height = 1000)**

**#save the model**

**save(xgb, file = "xgboost3.RData")**

**#load the model**

**load("xgboost3.RData ")**
